# Knotless Suture Anchors Display Favorable Elongation but an Inferior Ultimate Failure Load Versus Titanium Suture Anchors and All-Suture Anchors: A Biomechanical Comparison in a Porcine Model

**DOI:** 10.1177/23259671241300520

**Published:** 2024-12-13

**Authors:** Adrian Deichsel, Jana Rolf, Michael J. Raschke, Alexander Milstrey, Matthias Klimek, Christian Peez, Elmar Herbst, Christoph Kittl

**Affiliations:** * Department of Trauma, Hand and Reconstructive Surgery, University Hospital Münster, Münster, Germany; Investigation performed at the Department of Trauma, Hand and Reconstructive Surgery, University Hospital Münster, Münster, Germany

**Keywords:** suture anchors, knotless, all-suture, medial collateral ligament, repair

## Abstract

**Background:**

Several types of suture anchors, which differ in their working principles, are available for fixation of ligamentous structures in knee surgery. How the choice of a suture anchor type influences the biomechanical stability of ligament fixation is largely unknown.

**Purpose:**

To compare the biomechanical properties of different suture anchor designs regarding primary stability for tendon fixation and repair in medial collateral ligament (MCL) surgery.

**Study Design:**

Controlled laboratory study.

**Methods:**

The primary stability of MCL fixation was assessed in a porcine model utilizing 1 of 3 suture anchor types: a 5.5-mm titanium suture anchor (TSA), a 2.8-mm all-suture anchor (ASA), or a 5.5-mm polyether ether ketone knotless suture anchor (KLSA). Primary stability was assessed using a uniaxial material testing machine. Cyclic loading at 50 N and 100 N was applied for 500 cycles each, followed by a load-to-failure test.

**Results:**

After 500 cycles at 50 N, the KLSA (2.4 ± 0.3 mm) showed significantly (*P* < .05) reduced elongation in comparison to the TSA (4.0 ± 0.9 mm) and ASA (3.6 ± 0.7 mm), and after 500 cycles at 100 N, the KLSA (6.5 ± 1.4 mm) again showed significantly (*P* < .05) reduced elongation in comparison to the TSA (11.0 ± 2.2 mm) and ASA (12.0 ± 3.6 mm). However, the KLSA (213 ± 27 N) showed a significantly (*P* < .05) inferior ultimate failure load in comparison to the TSA (300 ± 20 N) and ASA (348 ± 23 N). In comparison to the TSA (113.0 ± 11.0 N/mm) and ASA (113.6 ± 14.4 N/mm), the KLSA (150.7 ± 11.6 N/mm) displayed the highest stiffness (*P* < .05). No significant differences were observed regarding yield load.

**Conclusion:**

KLSAs displayed significantly reduced elongation, at the cost of a reduced ultimate failure load, in comparison to TSAs and ASAs.

**Clinical Relevance:**

Surgeons should be aware that differences exist between different suture anchor types regarding their biomechanical stability. KLSAs may be favorable for fixation of peripheral ligaments in knee surgery.

An injury to the medial collateral ligament (MCL), either in isolation or concomitant with a tear of the anterior cruciate ligament (ACL), is described as one of the most frequent ligament injuries of the knee.^5^ While most isolated MCL injuries can be treated nonoperatively, combined injuries with high-grade valgus instability are eligible for surgical treatment of the torn MCL in combination with ligament surgery.^13,42^ Because most MCL tears occur near the femoral attachment, and in fewer cases near the tibial insertion site, repair of the detached MCL is advocated by several authors.^19,23,30,37,49^ The choice of an implant for repair is limited because the ligament must be approximated to the insertion site, making implants such as interference screws or bone staples unsuitable. Therefore, repair is frequently, but not exclusively, performed using suture anchors.^19^ For reconstruction with free grafts, several other fixation modalities such as interference screws, staples, and suture buttons are additionally available.

Several suture anchor types are available for fixation of ligamentous structures in knee surgery, loaded with high-strength polyethylene sutures.^53^ However, the method of fixation to the bone differs between the different types of suture anchors. Conventional suture anchors consist of an anchor body, made from titanium or a biodegradable material, which are inserted (typically screwed) into the bone and contain ≥1 sutures that are used for fixation of soft tissue structures.^18^ All-suture anchors (ASAs) use a sleeve or tape, made from suture material, through which the sutures are woven. When an ASA is inserted into the bone and the main suture is pulled back, the radiolucent sleeve expands and blocks behind the cortical bone, serving as fixation of the sutures to the bone, which allows fixation through a smaller cortical hole.^32^ Finally, knotless suture anchors (KLSAs) allow fixation of the suture to the bone without the need of knot-tying.^50^ Fixation is typically performed by whipstitching soft tissue first, followed by shuttling of the sutures through an eyelet at the tip of the suture anchor, which is then inserted into a predrilled bone hole to secure the suture.^53^

While originally developed for shoulder surgery, all of the mentioned implants are currently also used for fixation of ligaments in surgery of peripheral ligamentous structures of the knee.^20,44,52^ However, how the choice of a suture anchor type influences the primary stability (mechanical stability of fixation at the time of implantation) of ligament fixation in peripheral knee surgery is largely unknown. Therefore, the purpose of this biomechanical study was to compare a titanium suture anchor (TSA), ASA, and KLSA to assess which implant type might be biomechanically most stable for MCL fixation. It was hypothesized that because knots, which can elongate, need to be tied with a TSA and ASA, a KLSA would elongate less than a TSA and ASA. It was furthermore hypothesized that an ASA would show a comparable load to failure as a TSA.

## Methods

No ethics approval for this study was required by our institutional review board, and all implants and materials used in this study were commercially purchased. A porcine model of MCL fixation was performed in 2022 using 1 of 3 different suture anchor types. Porcine shanks were obtained from a local butcher, who confirmed the adequate health and absence of injuries of all animals used. An a priori power analysis showed that a sample size of 10 specimens per group would yield 90% power to detect a difference of 50 N between group means at the *f*≥ 0.8 level based on the standard deviations of graft fixation methods in porcine knee models of prior studies.^17,40^

### Suture Anchor Fixation

**Figure 1.**
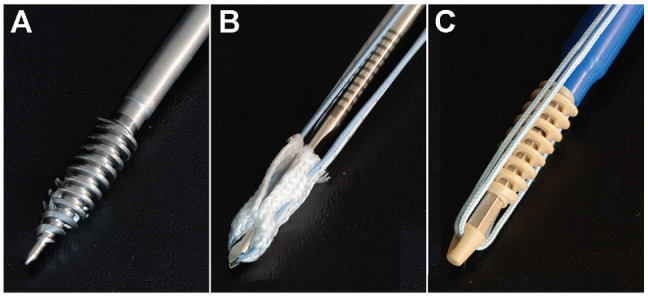
Suture anchor types used in this study. (A) A 5.5-mm titanium suture anchor (Twinfix Ultra with No. 2 Ultrabraid; Smith & Nephew). (B) A 2.8-mm all-suture anchor (Y-Knot PRO RC with No. 2 Hi-Fi; ConMed). (C) A 5.5-mm polyether ether ketone knotless suture anchor (SwiveLock with No. 2 FiberWire; Arthrex).

The 3 suture anchor designs used in this study were as follows (n = 10 each):

- 5.5-mm TSA (Twinfix Ultra with No. 2 Ultrabraid; Smith & Nephew),
- 2.8-mm ASA (Y-Knot PRO RC with No. 2 Hi-Fi; ConMed), and
- 5.5-mm polyether ether ketone (PEEK) KLSA (SwiveLock with No. 2 FiberWire; Arthrex) (Figure 1).

The tibia and superficial digital flexor tendon were prepared from fresh porcine hind legs and were frozen at −20°C. For testing, specimens were defrosted at 7°C for 24 hours and fixed in a cylindrical mount using synthetic resin (RenCast; Gößl & Pfaff). Superficial digital flexor tendons were harvested and trimmed to a length of 80 mm and a diameter of 6 mm to simulate the thickness of the human MCL.^16^ The anatomic tibial insertion site of the porcine MCL was found approximately 40 mm distal to the joint line and was exposed.^10^ In a randomized order, the suture anchors were inserted into the anatomic insertion of the MCL according to the manufacturer's instructions by a senior surgeon (C.K.). Predrilling a hole for suture anchor insertion was performed at diameters of 3.5 mm for the TSA, 2.3 mm for the ASA, and 4.5 mm for the KLSA. The distal 20 mm of the tendon graft was sutured with the Krackow technique utilizing the suture material of each tested suture anchor.^33^ With the TSA and ASA, 5 alternating throws of the surgeon's knot were used to secure the suture to the suture anchor. With the KLSA, the suture was shuttled into the bone tunnel, followed by screw-in of the anchor, pressing the sutures to the tunnel wall.

### Biomechanical Testing

**Figure 2.**
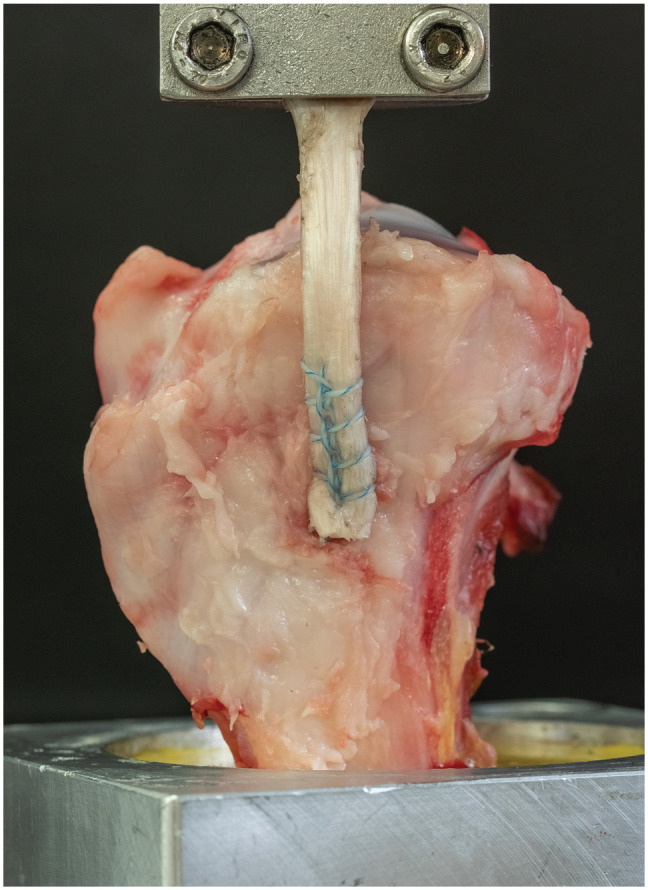
Experimental setup for biomechanical testing. Shown is a 5.5-mm titanium suture anchor affixed to a 6 mm–diameter tendon graft, sutured using the Krackow technique with high-strength polyethylene suture material to the anatomic MCL insertion site, approximately 40 mm distal to the joint line.

A servohydraulic uniaxial material testing machine (Model 8874; Instron) with a 0- to 20-kN sensor was utilized to determine biomechanical stability. The accuracy was ±0.005% (±1 N) of the load cells’ rated output, allowing position control with an accuracy of ±0.5% of the desired crossbar position. The cylindrical mount containing the embedded porcine tibia was fixed to the base of the machine with 2 clamps. The proximal end of the graft was fixed to the testing machine using a steel clamp with a serrated gripping surface,^26,54^ leaving 20 mm of the free graft between the clamp and the joint line (Figure 2).

Before testing was executed, the construct was manually pretensioned with a force of 20 N by positioning of the machine's crossbar. The following test protocol was applied, as previously described^10^: Before the start of cyclic loading, 10 cycles at 50 N were performed for preconditioning of the tendon graft. Cyclic loading was then performed at a frequency of 1 Hz. To simulate forces occurring in the native human MCL, 500 cycles at 50 N per cycle were performed to simulate forces in an ACL-intact knee, followed by 500 cycles at 100 N per cycle to simulate forces in an ACL-deficient knee.^35,36,46^ The different loads were performed to simulate the range of forces that repair of the MCL might be subjected to. Finally, the construct was continuously loaded at a speed of 25 mm/min until failure of the construct occurred.^8,9,41,51^

Stiffness was calculated from the slope of the linear portion of the load-displacement curve during load to failure. Yield load was defined as the point on the force-displacement curve at which elastic deformation ended and plastic deformation of the tendon/construct occurred (4%-6% of elongation).^24,31^ The mode of failure (pullout of suture or suture anchor, suture rupture) was macroscopically documented.

### Statistical Analysis

Data and statistical analyses were performed using MATLAB (Version R2020a; MathWorks) and Prism (Version 8; GraphPad Software). The criteria for normal distribution of the data were checked utilizing histograms as well as the Shapiro-Wilk test. Because not all groups passed the criteria of a Gaussian distribution, the Kruskal-Wallis test was used. The post hoc Dunn test was performed to adjust for multiple comparisons. A *P* value of <.05 was deemed to identify significant differences.

## Results

**Figure 3.**
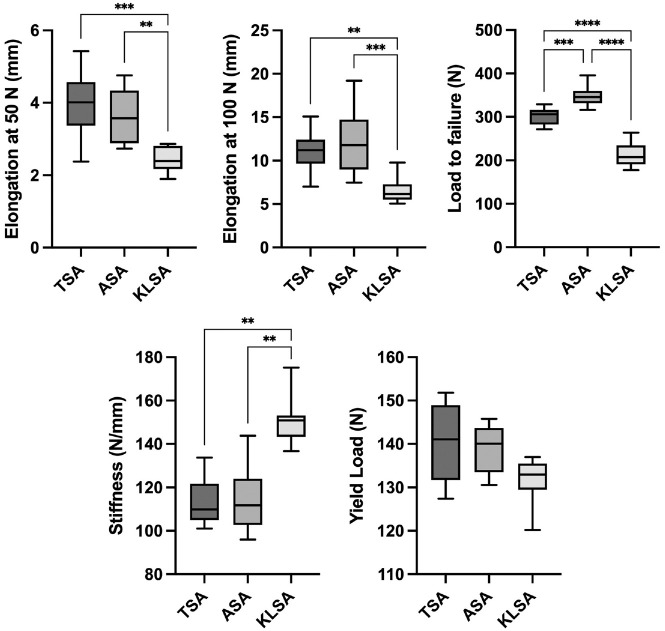
Elongation after cyclic loading at 50 N and 100 N, load to failure, stiffness, and yield load as boxplots presented as the mean, standard deviation, and range. ASA, all-suture anchor; KLSA, knotless suture anchor; TSA, titanium suture anchor. *P≤ .05. **P≤ .01. ***P≤ .001. ****P≤ .0001.

After 500 cycles at 50 N, the KLSA group (2.4 ± 0.3 mm) showed significantly less elongation compared to the TSA (4.0 ± 0.9 mm; *P* < .001) and ASA (3.6 ± 0.7 mm; *P* < .01) groups (Figure 3 and Table 1). After 1000 cycles of loading (500 cycles at 50 N and 500 cycles at 100 N), the KLSA group (6.5 ± 1.4 mm) showed significantly less elongation in comparison to the TSA (11.0 ± 2.2 mm; *P* < .01) and ASA (12.0 ± 3.6 mm; *P* < .001) groups. The load to failure was 300 ± 20 N in the TSA group, 348 ± 23 N in the ASA group, and 213 ± 27 N in the KLSA group. The KLSA group showed a significantly lower ultimate failure load in comparison to the TSA (*P* < .05) and ASA (*P* < .0001) groups.

**Table 1:** Results of Primary Stability Testing^*a*^

	TSA Group	ASA Group	KLSA Group
Elongation at 50 N, mm	4.0 ± 0.9	3.6 ± 0.7	2.4 ± 0.3
Elongation at 100 N, mm	11.0 ± 2.2	12.0 ± 3.6	6.5 ± 1.4
Load to failure, N	300 ± 20	348 ± 23	213 ± 27
Stiffness, N/mm	113.0 ± 11.0	113.6 ± 14.4	150.7 ± 11.6
Yield load, N	141.0 ± 8.7	139.0 ± 5.5	131.0 ± 5.3

a Data are reported as mean ± SD. ASA, all-suture anchor; KLSA, knotless suture anchor; TSA, titanium suture anchor.

Stiffness was 113.0 ± 11.0 N/mm in the TSA group, 113.6 ± 14.4 N/mm in the ASA group, and 150.7 ± 11.6 N/mm in the KLSA group. The KLSA group showed significantly higher stiffness in comparison to the TSA (*P* < .01) and ASA (*P* < .01) groups.

The yield load was 141.0 ± 8.7 N in the TSA group, 139.0 ± 5.5 N in the ASA group, and 131.0 ± 5.3 N in the KLSA group. There was no significant difference observed in the yield load between the different fixation constructs. The mode of failure was suture rupture in all specimens in the TSA and ASA groups and pullout of the suture from the bone tunnel in all specimens in the KLSA group.

## Discussion

The most important finding of this study was that the KLSA showed significantly less elongation after extensive cyclic loading, in comparison to the TSA and ASA, for tibial fixation of an MCL graft. Furthermore, the ASA, although smaller in diameter (2.8 vs 5.5 mm, respectively), was able to withstand comparable loads in comparison to the TSA. Recently, surgical treatment with repair of femoral or tibial avulsions has been advocated for improved outcomes of acute high-grade MCL lesions, with a suture anchor being the implant of choice.^19,21^ However, MCL repair utilizing suture anchors was also reported to lead to increased failure rates and reduced knee stability in comparison to MCL reconstruction.^2^ In addition to several other factors, the choice of an implant for repair might be a contributing factor to this phenomenon. Based on the results of the present study, surgeons should be aware that differences exist between different suture anchor types regarding their biomechanical stability. How these findings translate into clinical reality is, however, unclear.

Although the KLSA displayed significantly less elongation than the TSA and ASA, the load to failure was significantly inferior to the other groups. When investigating the biomechanical stability of implants for ligament fixation, the ultimate failure load is typically reported as the primary outcome of interest, with multiple studies reporting solely this outcome.^39,53^ Contrarily, we feel that elongation under cyclic loading should also be considered as an important outcome when evaluating the performance of an implant for ligament and tendon fixation. During surgery, the tension of the fixed structure is adjusted to resemble physiological conditions as closely as possible. If an implant allows too much elongation, this tension is lost, leading to excessive laxity of the joint. A 3-mm joint space opening has been suggested as a threshold to distinguish between MCL-intact and MCL-deficient knees.^22,27^ In the present study, only the KLSA was able to stay below this threshold during cyclic loading at 50 N. However, it has to be stated that forces acting on the MCL during rehabilitation might be lower than the loads used in this study because of limited weightbearing in the initial phase after surgery.^14,45^ Under cyclic loading at 100 N, all tested suture anchors showed extensive elongation, with >5-mm elongation for the KLSA and >10-mm elongation for the TSA and ASA, suggesting the failure of all constructs at higher loads. When looking at load to failure, all suture anchor types were able to withstand loads, which are present during ACL rehabilitation.^36^ Furthermore, the yield load of all 3 tested implants was comparable. The clinical relevance of the observed differences in stiffness is unclear.

The superiority of the KLSA regarding elongation in this study may be explained by the absence of a knot for fixation of the suture to the suture anchor. Tied suture knots are known to elongate under cyclic loading.^28,34^ In a biomechanical evaluation, utilizing a material testing machine, loads of 21.9 to 58.8 N were necessary to elongate suture loops with different knot types and suture materials by 3 mm, which was defined as biomechanical failure.^25^ By removing the need to tie a knot over the suture anchor, elongation of the construct may be reduced. Indeed, in a previous biomechanical study utilizing a material testing machine for examining suture anchors in polyurethane bone blocks, knotless fixation of a No. 2 polyethylene suture was shown to lead to a higher load until biomechanical failure (3-mm construct elongation), as well as increased stiffness, in comparison to a knotted construct.^11^

Different generations and variations of suture anchors have been compared, as well as to other fixation modalities, in several biomechanical studies regarding their primary stability. As an example, Barber and Herbert,^3^ in their biomechanical study, investigated different iterations of an ASA, utilizing a material testing machine and synthetic bone blocks, and found that an ASA with a smaller diameter withstood reduced ultimate failure loads in comparison to that with a larger diameter, with the 2.8-mm Y-Knot (ConMed) suture anchor, as used in the present study, displaying the highest ultimate failure load (602.9 ± 159.0 N) if 3 No. 2 sutures were used for fixation. Elongation ranged from 1.39 to 3.52 mm, in contrast to 12.0 ± 3.6 mm in the present study. However, Barber and Herbert^3^ performed cyclic loading with only 200 cycles and extensive preloading, which might explain the differences between the studies. Another biomechanical study comparing an ASA with an interference screw in a human cadaveric shoulder model of subpectoral biceps tenodesis (500 cycles at 70 N) found comparable ultimate failure loads (239 vs 254 N, respectively) but significantly increased elongation (8.1 vs 3.4 mm, respectively).^7^ Another study compared different extracortical fixation strategies for MCL reconstruction in a porcine model, similar to the one used in the present study (250 cycles at 100 N), finding increased elongation when using a single (11.9 ± 5.2 mm) or double (8.3 ± 1.6 mm) TSA in comparison to a cancellous screw with a spiked PEEK washer (2.9 ± 0.7 mm).^40^ These results suggest that other fixation modalities (eg, suspension buttons, interference screws, staples) may provide increased primary stability for tendon fixation. However, these implants are better suited for ligament reconstruction rather than repair. Furthermore, adequate tendon healing to the bone after the use of suture anchors was reported in a large animal model of MCL repair,^48^ and clinically favorable results after ligament repair were reported,^29^ indicating the need for further studies in this area.

### Limitations

This biomechanical study had several limitations that should be mentioned. Notably, porcine knee anatomy and tendon biomechanics have been shown to be sufficiently similar to those of humans^12,43^ so that a porcine model can be used for orthopaedic implant research.^15-17,40^ However, the bone density in the porcine model is known to be significantly higher in comparison to humans,^1^ which could have biased the data in this study.^6,38^ A recent study showed that in a test setup similar to the one used in this study, the displacement of the machine actuator utilized to calculate elongation was significantly higher than actual graft slippage as measured by optical tracking at the tunnel aperture; however, both values were highly correlated.^4^ Therefore, the test setup used in this study was sufficient to compare different implant types. However, transfer to the clinical setting should be used with caution. As this was a time-zero study (biomechanical stability), the influence of the suture anchors on healing of the ligaments to the bone could not be evaluated. Furthermore, the suture anchors examined in this study utilized different high-strength suture materials. The differences in biomechanical properties of the suture materials could also contribute to the results of this study, independent of the suture anchor design.^47^

## Conclusion

In this study, KLSAs displayed significantly reduced elongation, at the cost of reduced ultimate failure loads, in comparison to TSAs and ASAs. Surgeons should be aware that differences exist between different suture anchor types regarding their biomechanical stability.
